# Treatment of Herpes simplex encephalitis with the helicase-primase inhibitor pritelivir in an immunocompromised patient

**DOI:** 10.1038/s44259-026-00262-z

**Published:** 2026-09-02

**Authors:** Catherina Lueck, Christina Rautenberg, Alexander Birkmann, Susanne Bonsmann, Alessandra Marini, Melanie Sumner, Thomas Schroeder, Sebastian Voigt

**Affiliations:** 1 Department of Hematology and Stem Cell Transplantation, West German Cancer Center, University Hospital Essen, Essen, Germany; 2 AiCuris Anti-Infective Cures AG, Wuppertal, Germany; 3 Institute for Virology, University Hospital Essen, University of Duisburg-Essen, Essen, Germany

**Keywords:** Diseases, Immunology, Medical research, Microbiology, Neurology, Neuroscience

## Abstract

Pritelivir, a novel helicase-primase inhibitor, has successfully completed a global Phase 3 trial for refractory herpes simplex virus (HSV) infections in immunocompromised patients. This case report is the first clinical use of pritelivir in Herpes simplex encephalitis (HSE). An allogeneic hematopoietic cell transplant recipient developed HSE and was treated with acyclovir. After 1.5 months, while still on valacyclovir prophylaxis, HSE relapsed, confirmed by Magnetic Resonance Imaging (MRI) and HSV1 DNA detection in cerebrospinal fluid (CSF). Following transient response to acyclovir and foscarnet, combined pritelivir/acyclovir treatment was initiated in this comatose patient with cerebral edema and necrosis. Clinical improvement occurred with increased alertness and motor activity, enabling discharge to rehabilitation. MRI showed no further progression, and CSF HSV DNA load became undetectable. No relevant adverse effects were observed. Pritelivir plasma and CSF concentrations exceeded the 50% effective concentration for HSV1. This case highlights the potential of pritelivir for treating HSE.

## Introduction

Acute herpes simplex encephalitis (HSE) is a rare disease with an incidence of 0.2–0.3/100,000/year^1^. However, with its necrotizing hemorrhagic pathophysiology, HSE has a lethality of approximately 30% despite adequate treatment and causes permanent neurological damage in up to 70% of the patients^2^. HSE is caused by herpes simplex virus (HSV) type 1 in the vast majority (> 95%) of cases^1^ and manifests as either primary infection or reactivation^3^.

Recipients of allogeneic hematopoietic cell transplantation (HCT) are at high risk of HSV reactivation; nevertheless, encephalitis remains a rare complication, affecting approximately 0.1% to 1.8% of all HCT recipients^4,5^. Standard treatment for HSE is high-dose intravenous acyclovir (3 × 10 mg/kg/d). In case of clinically refractory or laboratory-proven acyclovir resistance, foscarnet serves as the alternative with effective cerebrospinal fluid (CSF) penetration^6–8^.

Pritelivir is an oral helicase primase inhibitor that has demonstrated potent antiviral activity in vitro, in vivo, and in patients with genital herpes^9^. Pritelivir was granted Breakthrough Therapy Designation by the U.S. FDA. A Phase 3 global trial in immunocompromised patients with refractory HSV infection, defined as not responding to at least 7 days of appropriately dosed anti‑HSV therapy^10^, has been completed (NCT03073967). Pritelivir achieved a clinically meaningful and statistically significant superiority in lesion healing compared to investigator’s choice of treatment^11^. Pritelivir is accessible through an early access program (EAP) for immunocompromised patients with acyclovir-refractory or resistant infections that are also foscarnet-refractory or intolerant (NCT05844436). In a mouse model of HSE, pritelivir demonstrated promising efficacy against acyclovir-resistant HSV strains, while combination therapy with acyclovir (acyclovir 10 mg/kg, pritelivir 0.1–0.3 mg/kg) in acyclovir-sensitive infections resulted in enhanced antiviral activity, suggesting additive or synergistic effects^12^.

## Results

### Case report

Here, we report the first clinical use of pritelivir after failure of acyclovir and foscarnet in a patient with HSE. The patient was a 49-year-old man who received HCT for treatment-related acute myeloid leukemia after multiple myeloma therapies. He tested positive for anti-HSV1 IgG antibodies before HCT. After conditioning with fludarabine and treosulfan, he received an immunosuppressive treatment with antithymocyte globulin, tacrolimus, and mycophenolate mofetil. The initial post-transplant course was uncomplicated with good hematologic engraftment, complete cytologic and molecular remission, and no clinical signs of acute graft-versus-host disease.

**Fig. 1:**
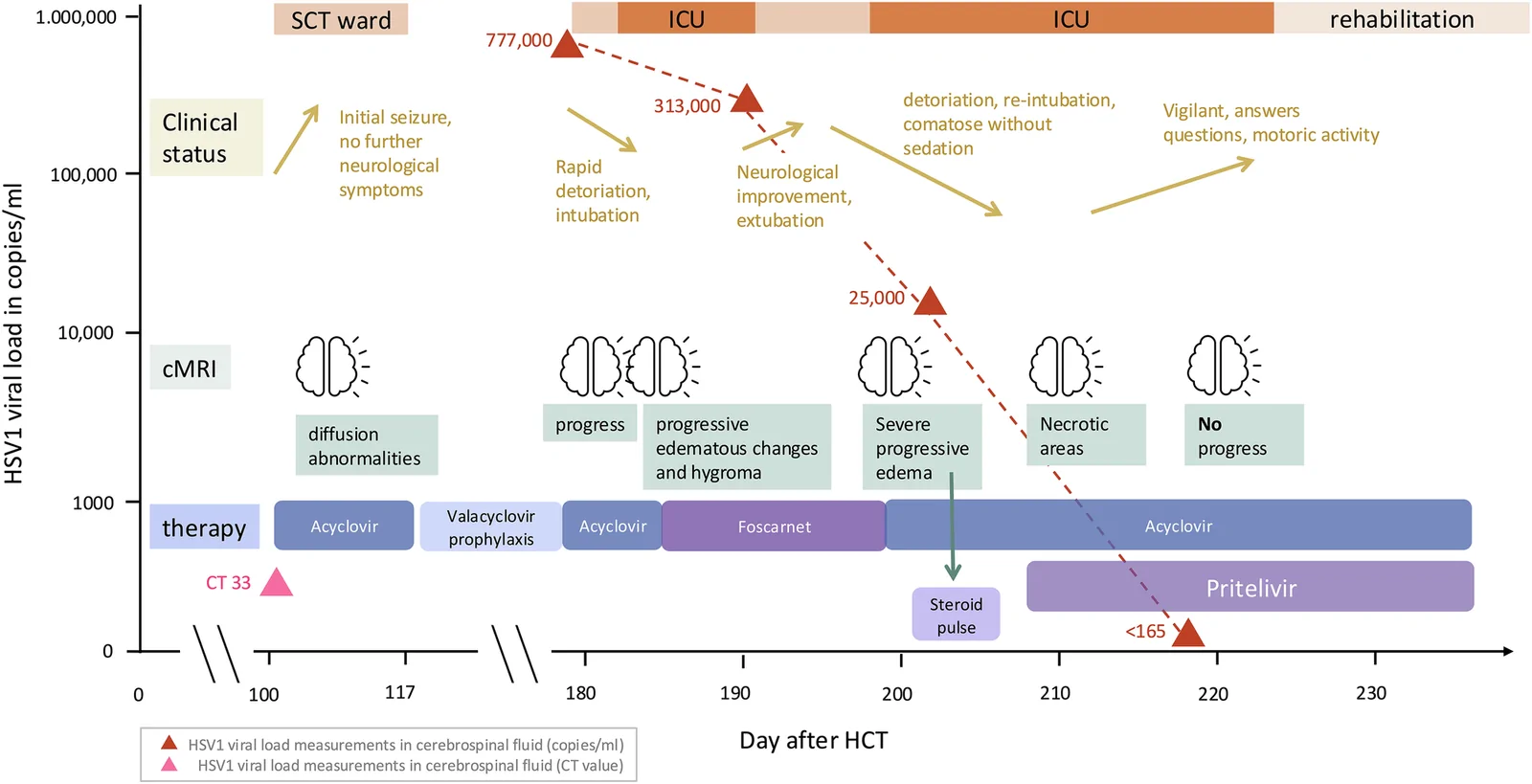
Time course (days after HCT) of HSV1 viral loads, antiviral treatment, cMRI exams, clinical status, and inpatient stays are shown. Viral loads (copies/ml) of HSV1 were measured in the cerebrospinal fluid. A dashed red line depicts the progress of HSV1 viral loads.

On day +102 after HCT, he developed a tonic-clonic generalized seizure. The computed tomography (CT) scan showed no acute pathology. The CSF cell count was borderline elevated at 5 cells/µl, and a multiplex PCR encephalitis panel (FTD Neuro9 (Fast Track Diagnostics, Siemens Healthineers)) was positive for HSV1, with a low-to-moderate cycle threshold of 33, despite valacyclovir prophylaxis. Cerebral magnetic resonance imaging (cMRI) showed no barrier dysfunction, but hyperintense right temporomesial changes and bilateral insular and frontobasal changes with diffusion abnormalities. Treatment with acyclovir (3 x 10 mg/kg i.v. per day) was initiated, and intravenous immunoglobulin was replaced (Fig. 1). The patient received levetiracetam to prevent further seizures. He did not experience any additional neurological disturbances and was discharged after 14 days of intravenous acyclovir treatment with subsequent valacyclovir (2 x 500 mg per day) secondary prophylaxis.

On day +178, while still on valacyclovir prophylaxis, the patient presented to the allogeneic HCT outpatient clinic with fever, cognitive impairment, and general weakness. cMRI showed progressive changes in the FLAIR (Fluid-Attenuated Inversion Recovery) sequence, which were accentuated in the right temporomesial, temporopolar, and bilateral frontobasal regions extending into the right basal ganglia and, via the anterior commissure, into the contralateral temporal lobe. Since the CSF contained HSV1 with 777,000 copies/ml (quantified by the Altona RealStar HSV PCR kit 1.0) and had over 200 cells/μl without signs of meningeal leukemia, the patient was restarted on intravenous acyclovir (3 × 10 mg/kg i.v. per day). However, he deteriorated and required intubation due to impaired consciousness five days after admission. Another cMRI was performed one week later that revealed ongoing progressive edematous changes and hygroma. As resistance to acyclovir was suspected, the antiviral treatment was switched to foscarnet (2 x 90 mg/kg/d for 14 days, followed by 2 x 30 mg/kg/d adjusted to kidney impairment). After three days on foscarnet, the patient improved neurologically and was extubated. While the patient remained disoriented, he was able to follow motor commands and answer simple questions. A lumbar puncture performed 13 days after the confirmed HSE relapse and 6 days after starting foscarnet showed a decrease in HSV1 viral load in the CSF to 313,000 copies/ml. After 15 days of intensive care, treatment was continued on the hematology ward.

However, the patient’s vigilance deteriorated thereafter. He developed facial paresis as well as paralysis of the left arm and leg, and was unable to communicate verbally. A follow-up cMRI showed severe progression with progressive edema of the right hemisphere. Resistance to acyclovir in the CSF could not be confirmed by UL23 genotyping, which identified polymorphisms C6G, G251C, P268T, Q89R, A192V, V267L, and D286E. We did not test for UL30 mutations in this case. As a result, we stopped foscarnet therapy and resumed intravenous acyclovir (2 × 10 mg/kg i.v. per day, renal adjusted). In addition, dexamethasone was started to treat the cerebral edema, and the clinical deterioration required intensive care readmission and reintubation. HSV1 remained detectable in CSF with 25,000 copies/ml. Despite several days of intravenous acyclovir therapy and discontinuation of sedatives, the patient remained unresponsive even to painful stimuli. As post-viral autoimmune encephalitis could not be excluded, a five-day course of methylprednisolone pulse therapy was added to the continuous antiviral treatment with acyclovir. While the patient remained conscious, there was no clinical improvement. A subsequent cMRI showed ongoing progress with necrotic areas.

As ultima ratio, the use of pritelivir was requested through the EAP. With the consent of the patient’s relative, he received pritelivir 100 mg daily via a nasogastric tube, following a 400 mg loading dose on the first day of treatment, in combination with intravenous acyclovir (2 x 10 mg/kg i.v. per day). Four days after initiation of therapy, we observed a clinical response with a steady increase in the patient’s level of vigilance. Six days after starting pritelivir therapy, the patient was able to answer simple, closed questions and showed prompt motor activity, initially on command and later spontaneously. No relevant adverse effects of acyclovir/pritelivir treatment were observed. Eight days after starting pritelivir therapy, the MRI showed no further progression for the first time since the HSE recurrence. The CSF HSV1 viral load fell below the limit of quantification four weeks after the start of pritelivir therapy. Bioanalytical evaluation revealed pritelivir concentrations of 3634 ng/mL in plasma and 43.5 ng/mL in CSF. The CSF concentration exceeded the pritelivir in vitro 50% effective concentration (EC_50_) of 10 ng/mL for HSV1 inhibition by approximately 4-fold^13^. Treatment with pritelivir was continued for 30 days. The patient was discharged to a neurological rehabilitation program. Unfortunately, he died five months later, on day +380 after HCT, due to long-term neurological sequelae.

## Discussion

This is the first documented clinical case of pritelivir in treatment-refractory HSE. This case highlights the potential role of pritelivir as a potent antiviral agent in the management of HSE, particularly in immunocompromised patients with refractory infection, and demonstrates that pritelivir crosses the blood-brain barrier and adequate CSF exposures can be achieved. Based on preclinical data in mice showing additive or potentially synergistic antiviral effects of combining pritelivir and acyclovir^12^, this suggests that the combination may have contributed to the observed clinical improvement, especially since no confirmed acyclovir resistance was detected. Further clinical data are needed to elucidate its therapeutic benefits and to determine whether earlier intervention could prevent long-term neurological sequelae.

## Methods

### Ethics and regulatory considerations

This retrospective case report describes the use of Pritelivir as an individual treatment attempt for a critically ill patient with refractory herpes simplex encephalitis. The use of Pritelivir in the Early Access Program was conducted in accordance with applicable national regulations governing compassionate use of investigational medicinal products and did not require separate ethics committee approval under the relevant legal framework.

Treatment was initiated based on an individualized risk–benefit assessment in the context of severe, life-threatening infection with limited therapeutic alternatives. In line with the principles of the Declaration of Helsinki, written informed consent was obtained from the patient’s legally authorized representative after comprehensive discussion of the experimental nature of the intervention, the limited clinical evidence regarding central nervous system efficacy, and potential risks and uncertainties.

## Supplementary information

## Data Availability

The data backing this case report's findings can be obtained from the corresponding author upon reasonable request. However, due to patient privacy and ethical considerations, the data are not publicly accessible.
